# P2X7 Receptor Inhibition Improves CD34 T-Cell Differentiation in HIV-Infected Immunological Nonresponders on c-ART

**DOI:** 10.1371/journal.ppat.1005571

**Published:** 2016-04-15

**Authors:** Inna Menkova-Garnier, Hakim Hocini, Emile Foucat, Pascaline Tisserand, Laure Bourdery, Constance Delaugerre, Clarisse Benne, Yves Lévy, Jean-Daniel Lelièvre

**Affiliations:** 1 INSERM U955, Institut Mondor de Recherche Biomédicale, Créteil, France; 2 Université Paris Est Créteil, Faculté de Médecine, Créteil, France; 3 Vaccine Research Institute, Créteil, France; 4 Laboratoire de Virologie Hôpital Saint-Louis, Paris, France; 5 Groupe Hospitalier Henri-Mondor Albert-Chenevier, Créteil, France; Vaccine Research Center, UNITED STATES

## Abstract

Peripheral CD4+ T-cell levels are not fully restored in a significant proportion of HIV+ individuals displaying long-term viral suppression on c-ART. These immunological nonresponders (INRs) have a higher risk of developing AIDS and non-AIDS events and a lower life expectancy than the general population, but the underlying mechanisms are not fully understood. We used an *in vitro* system to analyze the T- and B-cell potential of CD34+ hematopoietic progenitor cells. Comparisons of INRs with matched HIV+ patients with high CD4+ T-cell counts (immune responders (IRs)) revealed an impairment of the generation of T-cell progenitors, but not of B-cell progenitors, in INRs. This impairment resulted in the presence of smaller numbers of recent thymic emigrants (RTE) in the blood and lower peripheral CD4+ T-cell counts. We investigated the molecular pathways involved in lymphopoiesis, focusing particularly on T-cell fate specification (Notch pathway), survival (IL7R-IL7 axis) and death (*Fas*, *P2X7*, *CD39/CD73*). *P2X7* expression was abnormally strong and there was no *CD73* mRNA in the CD34+ cells of INRs, highlighting a role for the ATP pathway. This was confirmed by the demonstration that *in vitro* inhibition of the P2X7-mediated pathway restored the T-cell potential of CD34+ cells from INRs. Moreover, transcriptomic analysis revealed major differences in cell survival and death pathways between CD34+ cells from INRs and those from IRs. These findings pave the way for the use of complementary immunotherapies, such as P2X7 antagonists, to restore T-cell lymphopoiesis in INRs.

## Author Summary

Combined antiretroviral therapy (c-ART) has dramatically decreased AIDS-related mortality and morbidity. Nevertheless increased morbidity is still present in HIV+ patients namely among those who experience poor immune CD4+ T-cell restoration under c-ART (i.e. CD4 <500 cells/mm^3^). The mechanisms associated with poor immune restoration under c-ART remain poorly understood. Moreover for some patients insufficient immune restoration can be only seen as a time related issue. We showed an alteration of the capacity of hematopoietic precursors (CD34+) to differentiate into T cells in HIV+ patients with persistent low immune restoration despite long life treatment with c-ART. This impairment is associated with perturbation of the ATP pathway that may be targeted with specific therapies.

## Introduction

Combined antiretroviral treatment (c-ART) has greatly improved the outcome of HIV infection. The key objective of c-ART is to suppress viral replication and to induce the production of sufficient numbers of CD4+ T cells to prevent AIDS-defining (CD4+ T-cell counts below 200 cells/mm^3^), and non-AIDS-defining (CD4+ T-cell counts below 500 cells/mm^3^) severe events [1]. Immunological failure is defined as an inability to reach these levels of CD4+ T cells on c-ART (200 or 500 cells/mm^3^, depending on the type of event considered). In large cohort of patients displaying viral suppression, immunological success seemed to be largely time-dependent, as the number of CD4+ T cells seemed to increase steadily, even after seven years [2]. CD4+ T-cell restoration may be hindered by mechanisms related to HIV infection and its consequences, or modulated by host factors, both of which may affect T-cell homeostasis in the periphery or through effects on T-cell production. Demographic factors (age, sex, ethnic group [3–5]) affect CD4+ T-cell levels and, thus, immune restoration. The characteristics of HIV infection in the patient (CD4+ T-cell nadir, peak viral load, duration of infection and viral control on c-ART [4, 6–8]) are also key determinants of CD4+ T-cell recovery. Increases in immune activation [9, 10] and inflammation [11, 12] are currently considered to be the principal mechanisms underlying poor immunological responses on c-ART. Such alterations affect the homeostasis of the T-cell pool, modifying both peripheral and thymic T-cell levels [13]. Specific host genetic factors, including polymorphisms of genes of the inflammation/apoptosis pathway [14] or genes involved in T-cell development, such as *IL7R* [15], are also associated with poor CD4+ T-cell recovery.

Several studies have shown that HIV may affect CD34+ cells before they colonize the thymus to generate T lymphocytes [16–18]. It remains unclear whether these cells are directly infected [18–24], but the virus is widely thought to affect the microenvironment of the precursors and stromal cells in this organ [16, 17, 25, 26]. Many studies have linked persistent disturbances in CD34+ cells due to HIV infection with a decrease in the intrinsic clonogenic potential of these cells in humans [17, 20, 25, 27–35] and in simian models of infection [26, 36–38]. Some of these studies analyzed T-cell development during HIV infection [30, 31, 37–39], but only a few addressed this issue in the context of incomplete immune restoration [17, 34], and none focused on the specific impairment of T-cell development.

In this study, we observed a specific decrease in the T-cell potential of circulating CD34+ progenitors from patients displaying virological suppression on long-term c-ART but with poor CD4+ T-cell restoration. We also showed that CD34+ cells from INRs were extremely sensitive to the extracellular ATP pathway, as inhibition of the ATP receptor, P2X7, restored T-cell differentiation.

## Results

### Enrollment and characteristics of the patients

We selected, from our cohort of HIV+ patients, those with poor immunological CD4+ T-cell restoration (i.e. CD4+ T-cell count <500/mm^3^ and a CD4/CD8 ratio <1) and a plasma viral load that had remained below the detection threshold for more than eight years. These patients are referred to hereafter as “immunological non-responders” (INRs). Patients with high levels of immunological CD4+ T-cell recovery (i.e. with values close to those of the general population of uninfected individuals: >900 CD4+ T cells/mm^3^ and a CD4/CD8 ratio >1 [40]), referred to hereafter as “immunological responders” (IR), were selected and matched with INRs for factors predictive of immune recovery on c-ART, including estimated date of infection, treatment duration, periods with a sustained undetectable viral load (i.e. <50 HIV-1 RNA copies/mL), CD4+ cell nadir and pretreatment CD4+ counts (Table 1). Uninfected control individuals were matched with HIV-positive patients for age. Median (IQR) absolute CD4+ T-cell counts were, as expected, higher in HIV-positive IRs (1086 cells/mm^3^ (927–1194)) than in INRs (379.5 cells/mm^3^ (280.3–431); *P*<0.0001), but median absolute CD8+ counts did not differ between these two subsets of patients (644.5 cells/mm^3^ (568.5–798.5) for IRs and 621 cells/mm^3^ (492.5–808.8) for INRs; *P* = NS). We also assessed the viral reservoir, by measuring cell-associated HIV-1 DNA levels in peripheral blood mononuclear cells. IRs and INRs presented similar HIV-1 DNA levels (3.25 log_10_ HIV-1 DNA copies/mL (2.65–3.4) and 3.15 (2.75–3.36) respectively; *P* = NS).

**Table 1:** Characteristics of the subjects included in the study.

Characteristic	HIV-	HIV+ IR	HIV+ INR	*P*		
				HIV- vs IR	IR vs INR	HIV- vs INR
**Number**	18	16	16			
**Age *(year)***	47.5 (34–55.25)	46 (40.25–52.75)	53 (47–63)	*NS*	*NS*	*NS*
**Sex ratio, *F/M***	0.21	0.5	0.8	*NS*	*NS*	*NS*
**Ethnic origin, *%***						
**Caucasian**	NA	68.75	62.5		*NS*	
**African**	NA	31.25	37.5		*NS*	
**Infection duration *(years)***	-	10 (7.5–18.75)	13 (8–17)		*NS*	
**Treatment duration *(years)***	-	9.5 (7.25–14.75)	13 (8–16)		*NS*	
**Time for which the virus was undetectable *(years)*** ^a^	-	8 (6–14)	7 (6–12)		*NS*	
**CD4+ nadir *(cells/mm*** ^***3***^ ***)***	-	191 (103–261)	102 (48–197)		*NS*	
**VL at baseline *(copies/mL)*** ^b^	-	81040 (25529–500000)	46571 (16809–259102)		*NS*	
**CD4+ cell count at baseline *(cells/mm*** ^***3***^ ***)*** ^b^	-	225 (150–305)	191.5 (59.75–243.5)		*NS*	
**CD4+ cell absolute count *(cells/mm*** ^***3***^ ***)*** ^a^	NA	1086 (927–1194)	379.5 (280.3–431)		****	
**CD4+ cells, *%*** ^a^	49.13 (45.09–55.07)	41.85 (37.72–50.71)	27.46 (20.16–37.14)	*	***	****
**CD8+ cells, absolute count *(cells/mm*** ^***3***^ ***)*** ^a^	NA	644.5 (568.5–798.5)	621 (492.5–808.8)		*NS*	
**CD8+ cells, *%*** ^a^	19.85 (17.46–26.09)	23.64 (20.85–26.63)	36.82 (30.55–48.82)	*NS*	****	****
**CD4/CD8 ratio** ^a^	2.31 (1.8–3.02)	1.69 (1.39–1.86)	0.58 (0.48–0.77)	***	****	****
**CD19+ cells, %** ^a^	11.43 (8.79–14.81)	20.07 (10.29–28.56)	7.456 (4–17.08)	*NS*	*	*NS*
**HIV reservoir *(log***_***10***_ ***no*. *of HIV DNA copies/mL of plasma)*** ^a^ ^,^ ^c^	-	3.25 (2.65–3.4)	3.15 (2.75–3.36)		*NS*	

^a^ on entry into the study

^b^ at the time of c-ART initiation

^c^ HIV-1 2-LTR circles were quantified with primers spanning the 2-LTR circle junction, as previously described [41, 42].

Real-time PCR was performed in triplicate on each sample. HIV- = HIV-uninfected subjects; IR = immunological responders; INR = immunological non-responders; F = female; M = male; NA = not available. Medians (IQR) are shown. The Mann-Whitney and Kruskal-Wallis tests were used to compare two and three groups, respectively. Fisher’s exact test was used to compare sex ratios and ethnic origins. NS for *P*>0.05

*P<0.05

***P<0.001

**** *P*<0.0001.

### Impaired T-cell lymphopoiesis in INRs

**Fig 1:**
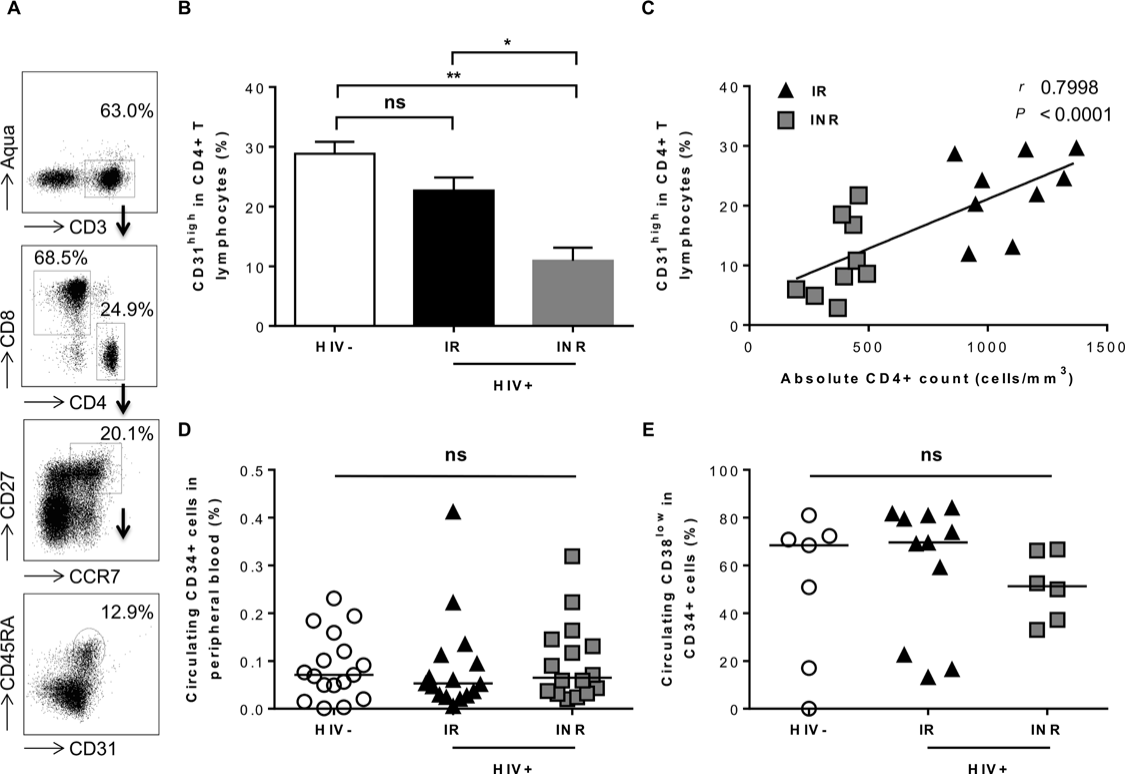
Analysis of lymphopoiesis in peripheral blood. (A) Gating strategy to identify recent thymic emigrants, RTEs. (B) Percentage of RTEs among CD4+ T lymphocytes in HIV-uninfected individuals (HIV-, *n* = 5), HIV+ IRs (*n* = 9) and INRs (*n* = 9). Mean values ± SEM are presented. The Kruskal-Wallis test was used for comparisons of three groups. NS for *P*>0.05, *P<0.05, **P<0.01. (C) Correlation between RTE frequencies and absolute CD4+ cell counts. Spearman’s rank correlation analysis was performed to determine the slope. ****P<0.0001. (D) Percentage of circulating CD34+ cells in the peripheral blood of HIV-uninfected subjects (HIV-, *n* = 18), HIV+ IRs (*n* = 16) and INRs (*n* = 16). Median values are shown. The Kruskal-Wallis test was used for comparisons of three groups, NS for *P*>0.05. (E) Percentage of CD38^low^ cells among circulating CD34+ cells in some HIV-uninfected subjects (HIV-, *n* = 7), IRs (*n* = 11) and INR (*n* = 6). Median values are shown. The Kruskal-Wallis test was used for comparisons of three groups, NS for *P*>0.05.

We analyzed the frequency of recent thymic emigrants (RTEs), defined as CD31^high^CD27+CCR7+CD45RA+CD4+ cells, in our three groups of subjects (Fig 1A). The percentages of RTEs among CD4+ cells were similar for HIV-uninfected individuals and IRs (28% (24.75–33.35) and 24.3% (16.7–29.05); *P* = NS; Fig 1B). By contrast, the frequency of CD4+ RTE cells was markedly lower in INRs (8.61% (5.46–17.65)) that in HIV-uninfected subjects and IRs (*P*<0.01 and *P*<0.05, respectively). The frequency of CD4+ RTEs was correlated with peripheral CD4+ cell count (*P*<0.0001; Fig 1C).

Levels of T-cell production may have been low due to less efficient seeding of the thymus by CD34+ cells. We therefore determined the frequency of CD34+ cells in peripheral blood. We found no difference in the frequency of these cells between the three groups (HIV-uninfected, 0.071% (0.0345–0.1395); IRs, 0.053% (0.0285–0.1085) and INRs, 0.0650% (0.03325–0.1415); overall *P* = NS; Fig 1D). The frequency of CD34+CD38^low^ immature progenitors in the blood was similar in HIV- individuals (68.43 (17–81)), IRs (69.64 (22.77–84.21)) and INRs (51.27 (36.16–66.67); overall *P* = NS; Fig 1E). Thus, INRs presented impaired T-cell lymphopoiesis, but had levels of circulating progenitors similar to those in IRs and in HIV- subjects.

### Alteration of T-cell differentiation potential of CD34+ cells is associated with poor CD4+ recovery

**Fig 2:**
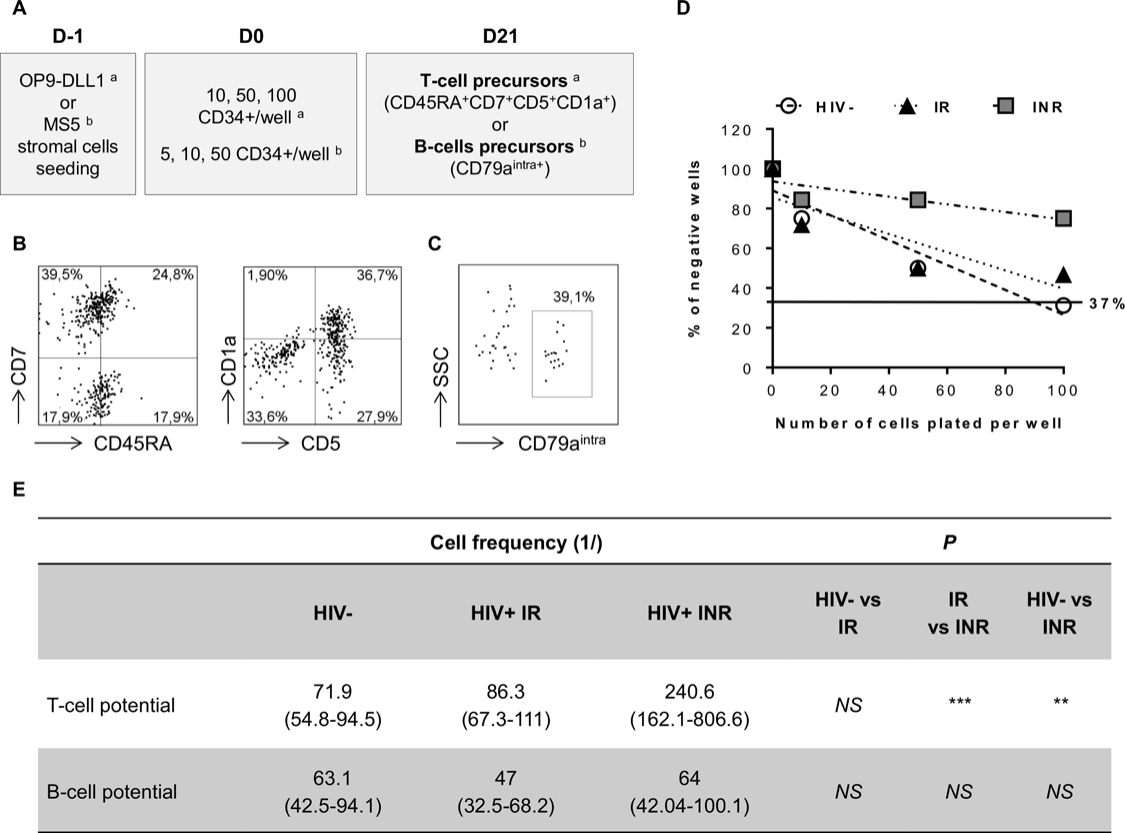
Limiting dilution assays (LDAs) to determine the T-cell and B-cell differentiation potential of CD34+ cells. (A) LDA design. Conditions for determining the potential of CD34+ cells to generate T cells ^a^ and B cells ^b^ are shown. For further details, see the materials and methods. (B, C) Cell cultures on D21 positive for T-cell precursors (B) defined as CD45RA^high^CD7+CD5+CD1a+ cells, and B cells (C) defined as CD79a^intra+^ cells. (D) Analysis of the T-cell potential of CD34+ cells. Each point on the graph represents the mean value from three independent experiments. (E) The presence of T-cell precursors and B cells was assessed with the ELDA webtool, applying the maximum likelihood method to the Poisson model. Mean values (min-max) for three experiments are indicated for each group and each set of conditions. NS for *P*>0.05, **P<0.01, ***P<0.001.

We then investigated whether the lower thymic output of INRs was due to alterations in CD34+ cell function. We assessed the lymphoid potential of circulating CD34+ cells in limiting dilution assays (LDAs; Fig 2A, 2B and 2D). The T-cell potential of CD34+ was similar in IRs and HIV-uninfected individuals (1/86.3 (1/67.3–1/111) vs. 1/71.9 (1/54.8–1/94.5); *P* = NS; Fig 2E). By contrast, this potential was much lower in INRs (1/240.6 (1/162.1–1/806.6)) than in either HIV-uninfected subjects (*P*<0.01) or IRs (*P*<0.001). We then investigated whether this alteration in the lymphoid potential of CD34+ cells was specific to the T-cell lineage. We performed LDAs in B-cell conditions (Fig 2A, 2C and 2E). We found no difference in the B-cell potential of CD34+ cells from HIV-uninfected subjects (1/63.1 (1/42.5–1/94.1)), IRs (1/47 (1/32.5–1/68.2)) and INRs (1/64 (1/42.04–1/100.1); overall *P* = NS). T-cell potential of these cells was inversely correlated with peripheral CD4+ T-cell counts and percentages in the patients studied (S1A and S1B Fig). Our results demonstrate that the T-cell differentiation potential of CD34+ cells is specifically altered in INRs and that this alteration is associated with poor CD4+ T-cell recovery on c-ART.

### The impairment of T-cell differentiation from CD34+ cells in INRs is not associated with altered responses to Notch or a particular genotype of *IL7RA*

**Fig 3:**
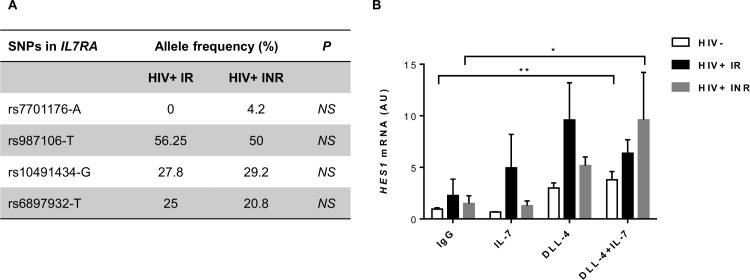
Analysis of IL7R polymorphisms and Notch activation in CD34+ cells from IRs and INRs. (A) *IL7RA* polymorphisms in HIV-infected patients. The allelic frequency of each SNP is shown. Fisher’s exact test was used to compare the distribution of SNPs between IRs (*n* = 9) and INRs (*n* = 10). NS for *P* >0.05. (B) Expression of the Notch target gene *HES1* in purified CD34+ cells from HIV-uninfected subjects (*n*≥3), IRs (*n* = 4) and INRs (*n* = 6) exposed overnight to IgG1-Fc (IgG), Delta-like 4 (DLL-4), and DLL-4 plus hIL-7 (DLL-4+IL7) (5 μg/mL for DLL-4 and 5 ng/mL for IL-7). The mean and standard error are shown. Kruskal-Wallis and Friedman tests were used to compare differences in mRNA levels between groups, for each set of conditions (unpaired data), and between sets of conditions for the same group (paired data), respectively. *P<0.05, **P<0.01.

The IL7/IL7R and Notch pathways are the two principal pathways of T-cell differentiation [43–47]. We therefore investigated whether perturbations of these two pathways could account for the lower T-cell potential of CD34+ cells in INRs. We assessed the prevalence in our patients of SNPs of the *IL7R* gene associated with decreases in T-cell production: SNPs present in the promoter region (rs7701176), exon 6 (rs6897932), intron 6 (rs987106) and the 3’ region (rs10491434) [15] (Fig 3A). There was no clear difference in the distribution of polymorphisms between the two groups of HIV-infected patients. Moreover, soluble *IL7RA* (S) and membrane-bound *IL7RA* (Mb) mRNA levels were similar in HIV-uninfected individuals and HIV-infected patients in studies on *ex vivo* purified CD34+ cells (overall *P*<0.05; S2A Fig). *NOTCH1* mRNA levels were also similar in subjects with and without HIV infection (overall *P*<0.05; S2B Fig). We assessed the functionality of the Notch1 receptor by analyzing the expression of Notch target genes in CD34+ cells after incubation with a recombinant Notch ligand, Delta-like 4 (hDLL4-Fc), in the presence of IL-7 [45]. *HES1* mRNA levels increased rapidly after incubation with DLL4 alone or together with IL-7, in cells from HIV-negative and HIV-infected individuals (*P*<0.05; Fig 3B). However, no difference in the expression of *HES1* was observed between IRs and INRs. These results suggest that alterations in Notch signaling and differences in *IL7RA* genetic background cannot account for the impaired lymphopoiesis in INRs.

### Persistent immune activation and inflammation in immunological nonresponders

**Fig 4:**
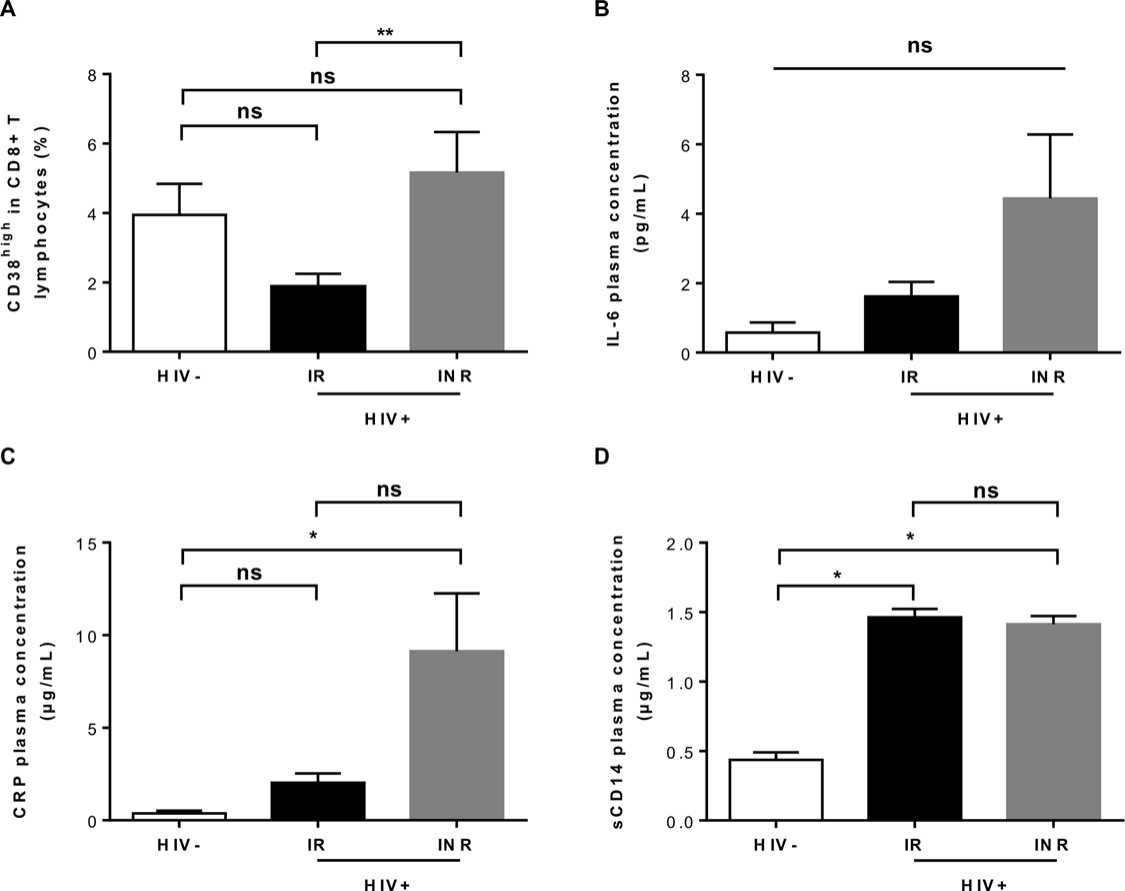
Immune activation and inflammation in HIV-uninfected individuals and HIV-infected patients. (A) Percentage of CD38^high^ cells among CD8+ T lymphocytes (HIV-, *n* = 18; IRs, *n* = 16; INRs, *n* = 16). (B, C, D) Plasma concentrations of IL-6 (B), CRP (C) and sCD14 (D) in HIV-uninfected (HIV-, *n* = 3) and HIV-positive individuals (IRs, *n* = 15; INRs, *n* = 16). Bars indicate the mean and standard error. The Kruskal-Wallis test was used to assess differences between groups. NS for *P*>0.05, *P<0.05, **P<0.01.

Chronic immune activation and inflammation have been shown to affect immune restoration in patients on c-ART [9–12, 48]. An analysis of the frequency of CD8+CD38+ T cells revealed very low levels of T-cell activation (1.25% (0.9863–2.755)) in IRs, similar to that in HIV-uninfected subjects (2.013% (1.452–6.6898); *P* = NS; Fig 4A). INRs had a higher percentage of CD8+CD38+ T cells than IRs (3.638% (2.509–7.28); *P*<0.01). However, CD8+CD38+ T-cell frequency in these patients was not correlated with CD4+ T-cell count or with the numbers of CD4+ RTEs in peripheral blood (*P* = NS; S3A and S3B Fig). Plasma IL-6 concentration was similar in HIV-uninfected subjects (0.76 pg/mL (0–0.96)) and IRs (1.06 pg/mL (0.56–2.04)), but tended to be slightly higher in INRs (1.7 pg/mL (1.01–3.78); overall *P* = NS; Fig 4B). Consistent with this observation, plasma CRP levels in INRs (3.45 μg/mL (0.58–9.21)) were higher than those in HIV-uninfected individuals (0.5 μg/mL (0.07–0.54); *P*<0.05), but not significantly different from those in IRs (1.16 μg/mL (0.75–3.19); *P* = NS; Fig 4C). Both IRs and INRs had higher plasma concentrations of sCD14 (1.45 μg/mL (1.36–1.57) for IRs and 1.37 μg/mL (1.19–1.62) for INRs; *P* = NS)) than HIV-uninfected subjects (0.48 μg/mL (0.33–0.5); *P*<0.05; Fig 4D). However, none of the soluble markers studied was positively correlated with peripheral CD4+ T-cell count in IRs and INRs (S4A, S4B and S4C Fig). Thus, INRs display persistent immune activation and inflammation despite virologically successful long-term c-ART.

### P2X7 is strongly expressed on CD34+ cells in INRs and its inhibition restores the potential for T-cell differentiation

**Fig 5:**
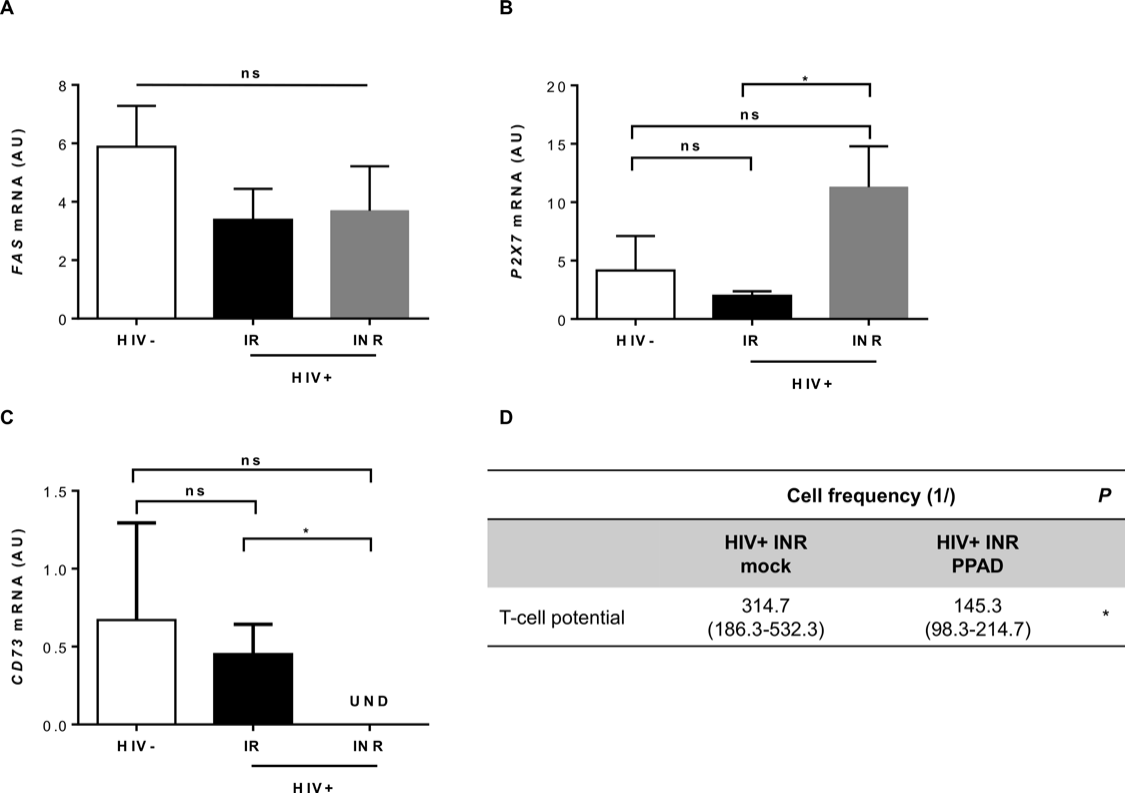
Molecular analysis of cell death pathways in CD34+ cells. (A, B, C) RT-qPCR analysis of *FAS* (A), *P2X7* (B) and *CD73* (C) mRNA levels in *ex vivo* purified CD34+ cells (HIV-, *n* = 6; HIV-positive IRs, *n* = 5; HIV-positive INRs, *n* = 4). Means and standard errors are shown. The Kruskal-Wallis test was used to assess differences between groups. NS for *P*>0.05, *P<0.05. (D) Limiting dilution assay, as described in Fig 2, to assess the potential to generate T cells, performed with CD34+ cells from HIV-positive INRs in T-cell medium alone (mock) or in T-cell medium supplemented with 20 μM PPAD. Mean (min-max) values for three patients are shown. The ELDA webtool was used. *P<0.05.

We then asked whether the impairment of CD34+ cell differentiation into T cells resulted from changes in death pathways due to persistent immune activation in INRs. We observed no difference in the *FAS* expression of CD34+ cells between HIV-uninfected subjects and HIV-infected IRs and INRs (Fig 5A, *P* = NS). Extracellular nucleotides and purinergic receptors modulate CD34+ cell homeostasis [49–53]. Among P2 family receptors *ex vivo* purified peripheral CD34+ express *P2X1*, *P2X4* and *P2X7* [54]. It is well documented that the binding of ATP to its receptor, P2X7, induces the assembly of a cytoplasmic multipartner complex, the inflammasome, and caspase-1 activation, leading to secretion of the proinflammatory cytokines IL1ß and IL-18 [55, 56]. We observed that *P2X7* was markedly more strongly expressed in INRs than in IRs and HIV-uninfected subjects (*P*<0.05; Fig 5B). Extracellular ATP may also be hydrolyzed by the ectoenzymes CD39/CD73 [57–62]. Particularly *CD73* expression was undetectable in all the INRs studied (*P*<0.05; Fig 5C).

We investigated the role of P2X7 in poor immune restoration in HIV patients, in LDAs involving irreversible antagonism with PPAD. We showed that P2X7 inhibition significantly improved the potential of CD34+ cells from INRs to differentiate into T cells (1/145.3 (1/98.3–1/214.7) in the presence of PPAD vs. 1/314.7 (1/186.3–1/532.3) in its absence; *P*<0.05; Fig 5D). The longitudinal follow-up of inflammation markers for up to four years in 15 HIV-positive patients showed no correlation with *P2X7* expression on CD34+ cells (S5A–S5F Fig). These results suggest that abnormally high levels of *P2X7* expression and an absence of *CD73* affect the normal differentiation of CD34+ cells into T cells in INRs, probably by increasing the susceptibility of these cells to extracellular ATP.

### Microarray analysis reveals a downregulation of cell survival pathways and an upregulation of apoptosis in CD34+ cells from INRs

**Fig 6:**
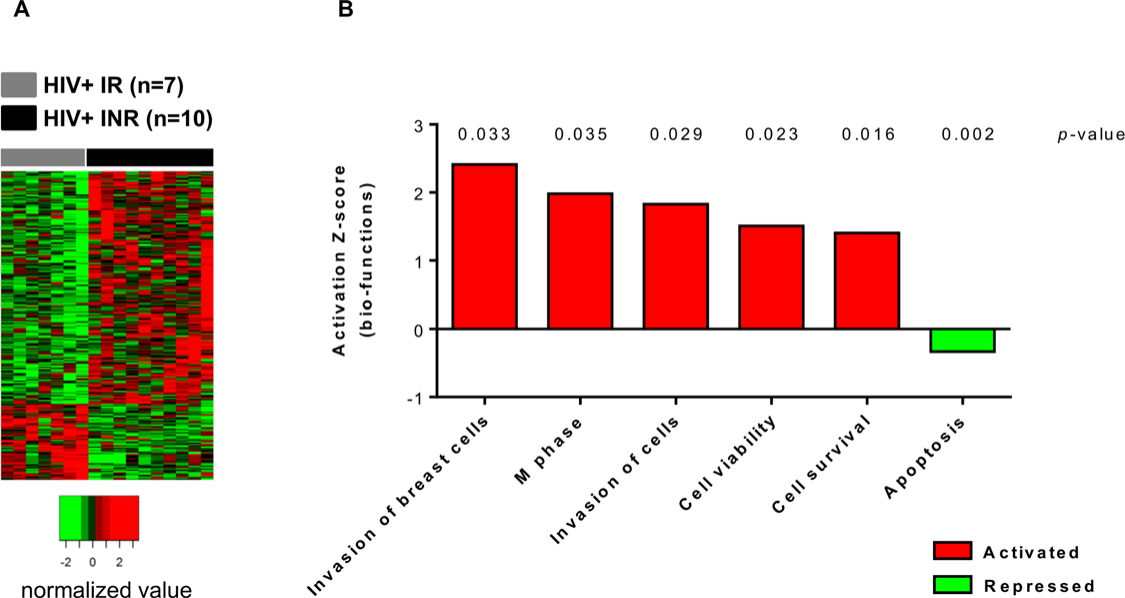
Transcriptomic analysis of CD34+ cells in HIV-infected IRs and INRs. (A) Genes differentially expressed in the CD34+ cells of IRs (*n* = 7) and INRs (*n* = 10) are shown. Each column represents an individual sample, and each row, an individual gene, with normalized expression level indicated on a color scale (red = upregulation, green = downregulation). (B) The top 5 biological functions in Ingenuity analysis, based on activation *z*-score, an algorithm predicting the degree of activation or inactivation of the genes of the group concerned. Rank in the top 5 is indicated by the number after the #. *P*-values are shown. Biological functions upregulated (positive *z*-score) in IR patients are shown in red, and biological functions downregulated in IR patients (negative *z*-score) are shown in green.

We explored the mechanisms underlying the functional alterations of CD34+ cells in INRs, by performing transcriptomic analysis in *ex vivo*-purified CD34+ cells from IRs and INRs (Fig 6A). The gene expression profiles of these two groups were very similar, with only 210 genes differentially expressed. These genes were grouped by biological function, and the top five were identified on the basis of activation *z*-score (Fig 6B). This score predicts the activated (positive *z*-score) or inactivated (negative *z*-score) state of genes from the same functional group. Cells from IR patients displayed an upregulation of genes from the following categories: mitosis M phase (*PKP4* (FC = 7.919), *SEPT11* (FC = 2.469)), cell viability (*DEF6* (FC = 1.64), *GADD45B* (FC = 1.752)) and survival (*PRKCZ* (FC = 3.104), *RFC1* (FC = 2.090)). In IRs, a negative *z*-score was attributed to apoptosis (*RNASEL* (FC = 6.642), *BIRC2* (FC = 2.375), *IFIH1* (FC = 1.939), *P*<0.01). Gene expression profile analysis revealed a role for general mechanisms of cell survival and death in CD34+ cells from INRs. The observed differences may underlie the low potential of these cells to differentiate into T cells and poor CD4+ T-cell recovery.

## Discussion

This study provides insight into the major mechanisms driving immune recovery in HIV-infected patients with long-term virological success on c-ART regimens. The design of this study differs from that of most other studies of poor immune recovery. First, we selected patients with opposite and extreme immunological profiles. Second, the patients enrolled in this study had been treated for at least eight years, and for up to 16 years in some cases, whereas most previous studies were carried out two to four years after c-ART initiation [4, 40, 63–66], with only a few investigating immune recovery mechanisms after more than five years of treatment [6, 67–69]. Finally, we applied strict criteria for patient selection, and most of the parameters predictive of immune recovery described in previous studies were characterized in our patients (age [3–5], sex [5, 70], and ethnic origin [70], durations of infection and treatment, duration of time for which the virus was undetectable, nadir and pre-therapy CD4+ counts [4, 6–8]).

Low levels of *de novo* lymphocyte production and peripheral dysfunction are considered to be major barriers to efficient immune restoration in HIV+ individuals [66, 71–75]. We showed, by assessing CD4+ RTE frequency, that lymphopoiesis was severely compromised in INRs. Our results strongly suggest that T-cell recovery is influenced by CD34+ cell impairment. We found no difference in peripheral CD34+ cell frequency between HIV-negative subjects, IRs and INRs. This finding is consistent with those of Mendez-Lagares *et al*. [65] and Baillou *et al*. [28], but conflicts with those of Sauce *et al*. [34]. We also found that the frequency of precursors already committed to T-cell development in IRs was no lower than that in INRs.

Finally, circulating CD34+ cells from INRs displayed functional alterations, with a specific decrease in T-cell precursor differentiation. Several studies have already reported a suppression of hematopoiesis in HIV-infected individuals [17, 20, 25, 27–35] and experimental animal models [26, 36–38], but only a few focused on T-cell development during HIV infection [30, 31, 34, 37–39]. One team used an indirect technique based on the use of surface markers expressed on circulating CD34+ cells to determine the lymphoid/myeloid ratio. They concluded that changes in lymphoid potential might be characteristic of HIV infection [34]. FTOC assays, a more appropriate analysis of T-cell development, revealed lower levels of immature (CD4+CD8+CD3-) and mature (CD4+CD8+CD3+) T-cell generation from blood CD34+ hematopoietic progenitor cells, regardless of the level of T-cell restoration [30, 31, 38]. However, FTOC provides no information about the frequency of T-cell precursors and cannot be used to quantify T-cell development. It may also be biased by the presence of mature contaminants. A similar impairment of hematopoiesis has been described in SIV infection [26, 36–38]. We cannot exclude the possibility that CD34+ cells from INRs also display impaired differentiation into other hematopoietic lineages. However, we observed no differences in B-cell generation between groups. Furthermore, we found that the potential of CD34+ progenitors to generate T cells was strongly correlated with the degree of peripheral T-cell restoration, implying that the maintenance of adequate lymphocyte levels is highly dependent on efficient *de novo* T-cell lymphopoiesis.

Some authors have suggested that CD34+ cells or more differentiated colonies may be directly infected [26, 28, 30, 36], regardless of the population studied, but there is little evidence to support this view. Several viral proteins (gp120 [76] or Nef [77]) have been shown to impair CD34+ differentiation. We quantified HIV RNA in culture supernatants, but detected no ongoing viral replication. We cannot rule out the possibility of latent infection, but the incidence of such infection would be too low to account for such specific changes in T-cell differentiation.

We investigated the molecular abnormalities underlying impaired lymphopoiesis, focusing, in particular, on the key factors for T-cell lymphopoiesis, Notch and IL7R. Some *IL7RA* SNPs have been reported to be associated with impaired immune recovery [14, 78, 79], but the prevalence of these SNPs was not higher in INRs than in other subjects, and no difference in the mRNA levels for soluble and membrane-bound IL7R were identified between groups. Target gene expression after a short period of Notch activation was similar in INRs and HIV-uninfected subjects. However, the timing of transcriptional programs downstream from Notch signaling is highly variable [80]. We cannot, therefore, exclude the possibility that some differences in the response to Notch ligands become visible only after longer periods of stimulation, although this would be difficult to test because survival issues affect mRNA quality at later time points in feeder cell-free culture systems.

Consistent with previous reports, we observed abnormal immune activation in INRs despite long-term c-ART. We also found that sCD14 levels remained high in treated HIV+ patients, as reported in a previous study [81]. Analyses of plasma samples collected from the HIV+ patients over the last few years showed that levels of soluble markers of inflammation remained stable and high over time. The observed inflammation would probably affect CD34+ cell survival. The Fas receptor did not seem to be involved in this process. We therefore investigated other cell death pathways involving ATP signaling. The availability of extracellular ATP is regulated by the CD39/CD73 ectoenzymes [57–62]. In the absence of ectonucleotidase activity, high ATP concentrations trigger the low-affinity P2X7 receptor to induce a massive release of proinflammatory cytokines, leading to cell death by pyroptosis [82]. We provide several lines of evidence suggesting that T-cell differentiation is impaired due to the extremely high sensitivity of CD34+ HPCs to extracellular nucleotides. CD34+ cells from INRs displayed *P2X7* upregulation and no *CD73* expression, suggesting a greater susceptibility of these cells than of those from other subjects to ATP-induced cell death. Consistent with this hypothesis, a P2X7 antagonist restored the T-cell differentiation of CD34+ cells in INRs. ATP has been reported to be involved in stem cell metabolism [49–53]. In murine hematopoietic and human neural progenitors, extracellular ATP causes rapid cell death and an increase in the frequency of apoptotic features [53, 83]. It is spontaneously released in cultures of human mesenchymal stem cells, inhibiting cell proliferation, and it appears to be a key regulator of early lineage commitment [84, 85]. Consistently, transcriptomic analysis revealed that, unlike the non-cycling cells of INRs, CD34+ cells from IRs underwent mitosis. The greater immune activation observed in the peripheral blood of INRs may also occur in the bone marrow. IL-6 and sCD14 are markers of the activation of monocytes/macrophages, the principal supporting cells of the bone marrow niche. LPS, through sCD14, may trigger TLR4-promoted cell death and initiate an auto-amplification loop in which dying cells release their contents, thereby creating a highly inflammatory environment for neighboring differentiating progenitors. In this setting, other P2X7-expressing cells may also contribute to the chronic inflammatory state. We observed no modification of the caspase-1 activation profile in hematopoietic progenitors *ex vivo*, suggesting that no pyroptosis was occurring. However, further studies are undoubtedly required to define the precise mechanism of CD34+ cell turnover in inflammatory conditions.

It also remains unclear why B-cell differentiation is unaffected. The role of IL7 in this process may provide an explanation. B-cell lymphopoiesis is dependent on IL7 in mice, but not in humans [86–88]. We have shown that the transcription of *P2X7* is increased by IL7 [89]. We did not measure plasma IL7 levels, but they are likely to be high in INRs, as either a cause or a consequence of the low CD4+ T-cell levels. We suggest that, at early stages of lymphopoiesis, IL7 potentiates extracellular nucleotide signaling, thereby inducing cell death and regulating stem cell metabolism. At late stages, the expansion of maturing progenitor populations is highly dependent on IL7 [45]. IL7 thus plays a dual role in T-cell development.

Finally, microarray comparisons of IRs and INRs revealed that CD34+ cells from INRs lacked transcripts associated with proliferation and survival. Transcript levels for *PKP4* and *SEPT11*, both of which are involved in cytokinesis during cell division [90, 91], and those for *PRKCZ*, an important target of PI3K signaling [92], *DEF6*, regulating lymphocyte survival [93], and *GADD45B*, involved in FOXO signaling, oxidative stress resistance and DNA repair [94], were also downregulated. Conversely, CD34+ cells from INRs displayed an upregulation of genes involved in cell death, such as *RNASEL* and *BIRC2* (encoding cellular inhibitor of apoptosis, cIAP) [95, 96]. Further characterization of the genes identified in microarray analyses should help us to determine which factors limit the differentiation into T cells of CD34 HPCs from INRs.

Our findings suggest that patients with limited immune recovery on c-ART could be given complementary treatments, such as anti-inflammatory compounds, and, more specifically, P2X7 inhibitors [97], which have been shown to have a good safety profile in clinical trials [98].

These findings provide compelling evidence that successful immune restoration in HIV-infected patients on c-ART involves the regeneration of new T cells from CD34+ cells. It may therefore be possible to identify potential targets for the enhancement of T-cell lymphopoiesis in patients with an incomplete restoration of CD4 T-cell counts on c-ART.

## Materials and Methods

### Patient samples

Peripheral blood samples were collected from HIV-negative healthy donors at the *Centre regional de transfusion sanguine* and from HIV+ patients on c-ART followed at Henri Mondor Hospital (Créteil, France). Ethics committee approval was obtained and the subjects included gave written informed consent, in accordance with the Helsinki Declaration, before the start of the study.

### Flow cytometry analyses

We used the following conjugated antibodies: CD38-fluorescein isothiocyanate, CD34-phycoerythrin (PE)-cyanin5 (Cy5), CD4-Pacific Blue, CD19-allophycocyanin (APC), CD3-Alexa Fluor 700, CD8-APC-H7, CCR7-Alexa Fluor 647, CD79a-APC from BD Biosciences (San Jose, CA); CD56-PE, CD45RA-PE-TexasRed, CD7-PE-Cy7, CD1a-PE, CD5-PE-Cy5 from Beckman Coulter (Brea, CA); CD31-PE from Miltenyi Biotech (Bergisch Gladbach, Germany) and CD27-PE-Cy7 from eBioscience (San Diego, CA). We obtained LIVE/DEAD aqua fluorescent dye from Molecular Probes (Invitrogen, Carlsbad, CA). The Fam-FLICA-FITC probe against active caspase-1 was used according to the manufacturers’ instructions (ImmunoChemistry, Bloomington, MN). Standard protocols were used for all types of staining. Data were acquired with an LSRII Flow Cytometer (BB Biosciences), and analyzed with FlowJo v7.6.5 (Treestar, Ashland, OR).

### Quantification of HIV-1 DNA and inflammation factors

Cell-associated HIV DNA was quantified by ultrasensitive real-time PCR (Biosentric, Bandol, France), in total PBMCs, as previously described [41]. Plasma samples were used to determine the concentrations of IL6, CRP and sCD14 with Quantikine ELISA kits (R&D Systems, Minneapolis, MN), according to the manufacturer’s protocol.

### Limiting dilution assay (LDA) for determining the potential of circulating CD34+ cells to generate T and B cells

PBMCs were separated on Ficoll-Hypaque gradients (PAA, Pasching, Austria). The CD34+ cells were isolated by immunomagnetic sorting with a Diamond CD34 Isolation Kit, according to the manufacturer’s instructions (Miltenyi Biotech). The CD34+ cell-enriched population had a purity >95%. We plated various numbers (10, 20, 50) of CD34+ cells on a layer of OP9-DLL1 stromal cells in the presence of 5 ng/mL hFlt3L and 5 ng/mL hIL-7 (R&D Systems) for T-cell development, or on MS5 stromal cells in the presence of IL-2 (10 ng/mL), IL-15 (1 ng/mL) and SCF (50 ng/mL) (Miltenyi Biotech) for B-cell development, as previously described [47]. Where specified, the P2X7 antagonist pyridoxal-phosphate-6-azophenyl-2’-4’disulfonate (PPAD) was added to the medium (20 μM, Sigma-Aldrich, St. Louis, MO). For limiting dilution assays, the cells were cultured for 21 days, then collected and analyzed for the presence of T-cell (CD45RA+CD7+CD5+CD1a+) or B-cell (CD79a^intra^) precursors. The frequency of T- and B-cell precursors was calculated with the ELDA webtool, by applying the maximum likelihood method to the Poisson model [47].

### Molecular analysis of mRNA levels

Purified CD34+ cells were exposed overnight to IgG1-Fc or DLL4-Fc (5 μg/mL, generously provided by A.Sakano [99]) in T-cell medium with or without IL-7, as previously described [45–47, 80]. RNA was isolated in TRIzol (Invitrogen), according to the standard procedure. RT-qPCR was carried out with the SuperScript VILO cDNA synthesis kit (Invitrogen) and Brilliant II SYBR Green Master Mix (Agilent, Santa Clara, CA), in standard conditions, on an MX3005P (Stratagene, La Jolla, CA). The primer sequences used have been described elsewhere [80].

### DNA extraction and sequencing

Total DNA was extracted from PBMCs collected from HIV-infected IRs and INRs, with the DNeasy Blood and Tissue Kit (Qiagen, Hilden, Germany) used according to the manufacturer’s protocol. PCR was performed with the Platinum *Taq* DNA Polymerase High Fidelity (Invitrogen), in standard conditions, on a 2720 Thermal Cycler (Applied Biosystems, Carlsbad, CA). The primers used to target SNPs were as follows: IL7RA-1 (3994–4281), IL7RA-2 (23356–23652), IL7RA-3 (25664–25969), and IL7RA-4 (22516–22811). The PCR products were sequenced in both directions with the BigDye Terminator v3.1 Sequencing Kit (Applied Biosystems) and processed on an automated sequencer (ABI PRISM 3130XL Genetic Analyzer, Applied Biosystems). Chromatograms were analyzed with Chromas (Technelysium, South Brisbane, Australia) and ClustalW (EMBL-EBI, Heidelberg, Germany).

### Microarray analysis

RNA was extracted from *ex vivo* purified CD34+ cells with the RNeasy Micro Kit (Qiagen), and quantified on an ND-8000 spectrophotometer (NanoDrop Technologies, Wilmington, DE). Its integrity was then checked on a 2100 BioAnalyzer (Agilent Technologies). *In vitro* transcription was performed on 60 ng of RNA (Ambion Illumina TotalPrep RNA Amplification Kits, Applied Biosystems/Ambion), as described elsewhere [100].

### Statistical analysis

All statistical analyses were performed with GraphPad Prism software v6 (La Jolla, CA). We used nonparametric Mann-Whitney and Kruskal-Wallis tests to compare continuous variables between two and three groups, respectively. For paired groups, Wilcoxon tests were used. Discrete variables were compared in Fisher’s exact tests. Differences were considered non-significant if *P*>0.05. Microarray data were analyzed by ViroScan3d (Lyon, France), as previously described [100]. Ingenuity pathway analysis (Qiagen) was also conducted, focusing on both canonical pathways and biological functions.

### Gene IDs mentioned in the text

*IL7RA/IL7R* (#3575), *NOTCH1* (#4851), *HES1* (#3280), *FAS* (#355), *P2X7/P2RX7* (#5027), *CD39/ENTPD1* (#953), *CD73/NT5E* (#4907), *PKP4* (#8502), *SEPT11* (#55752), *DEF6* (#50619), *GADD45B* (#4616), *PRKCZ* (#5590), *RFC1* (#5981), *RNASEL* (#6041), *BIRC2* (#329), *IFIH1* (#64135).

## Supporting Information

S1 Fig Correlation between T-cell potential and peripheral CD4+ cell levels. Correlation of T-cell potential with absolute CD4+ count (A) and the percentage of circulating CD4+ cells (B). Spearman’s rank correlation analysis was used to determine the slope. *P* values *<*0.05 were considered significant.
(TIF)

S2 Fig Molecular analysis of mRNA expression in *ex vivo* purified CD34+ cells. (A) RT-qPCR analysis of soluble (S) and membrane-bound (Mb) *IL7RA* (HIV-, *n* = 3; HIV-positive IRs, *n* = 10; HIV-positive INRs, *n* = 7). Means and standard errors are shown. The Kruskal-Wallis test was used to assess differences between groups and Wilcoxon test was used to compare sIL7RA to mbIL7RA for each group. NS for *P*>0.05. (B) RT-qPCR analysis of *NOTCH1* mRNA (HIV-, *n* = 4; HIV-positive IRs, *n* = 4; HIV-positive INRs, *n* = 5). Means and standard errors are shown. The Kruskal-Wallis test was used to assess differences between groups. NS for *P*>0.05.
(TIF)

S3 Fig Correlations of immune activation with peripheral absolute CD4+ count and CD4+ RTEs in HIV-positive INRs. (A, B) The percentage of CD38^high^ cells among CD8+ T lymphocytes indicates the degree of immune activation in correlation along peripheral absolute CD4+ T-cell count (A) and CD4+ RTEs percentage in peripheral blood (B). Spearman’s rank correlation analysis was used to determine the slope. *P* values <0.05 were considered significant.
(TIF)

S4 Fig Correlations of inflammation and peripheral absolute CD4+ count in HIV-positive INRs. (A) Plasma concentrations of IL-6 (A), CRP (B), and sCD14 (C) indicate the degree of inflammation. Spearman’s rank correlation analysis was used to determine the slope. *P* values <0.05 were considered significant.
(TIF)

S5 Fig Longitudinal analysis of soluble markers of inflammation and their correlation with *P2X7* expression. (A, B, C) Correlations of the mean concentrations of 8 available determinations per patient of IL-6 (A), CRP (B) and sCD14 (C) with *P2X7* mRNA levels (HIV-positive, *n* = 15). Spearman’s rank correlation analysis was used to determine the slope. *P* values <0.05 were considered significant. (D, E, F) Analysis of IL-6 (D), CRP (E) and sCD14 (F) concentrations over the last four years (2 time points per year) in 15 HIV-infected patients. Means and standard errors are shown. Friedman’s test was used for statistical analysis. *P* values <0.05 were considered significant.
(TIF)
