# Supplementary figures and images for: P2X7 Receptor Inhibition Improves CD34 T-Cell Differentiation in HIV-Infected Immunological Nonresponders on c-ART
Source: PLoS Pathog. 2016 Apr 15;12(4):e1005571. doi: 10.1371/journal.ppat.1005571 (PMC4833302; doi:10.1371/journal.ppat.1005571)

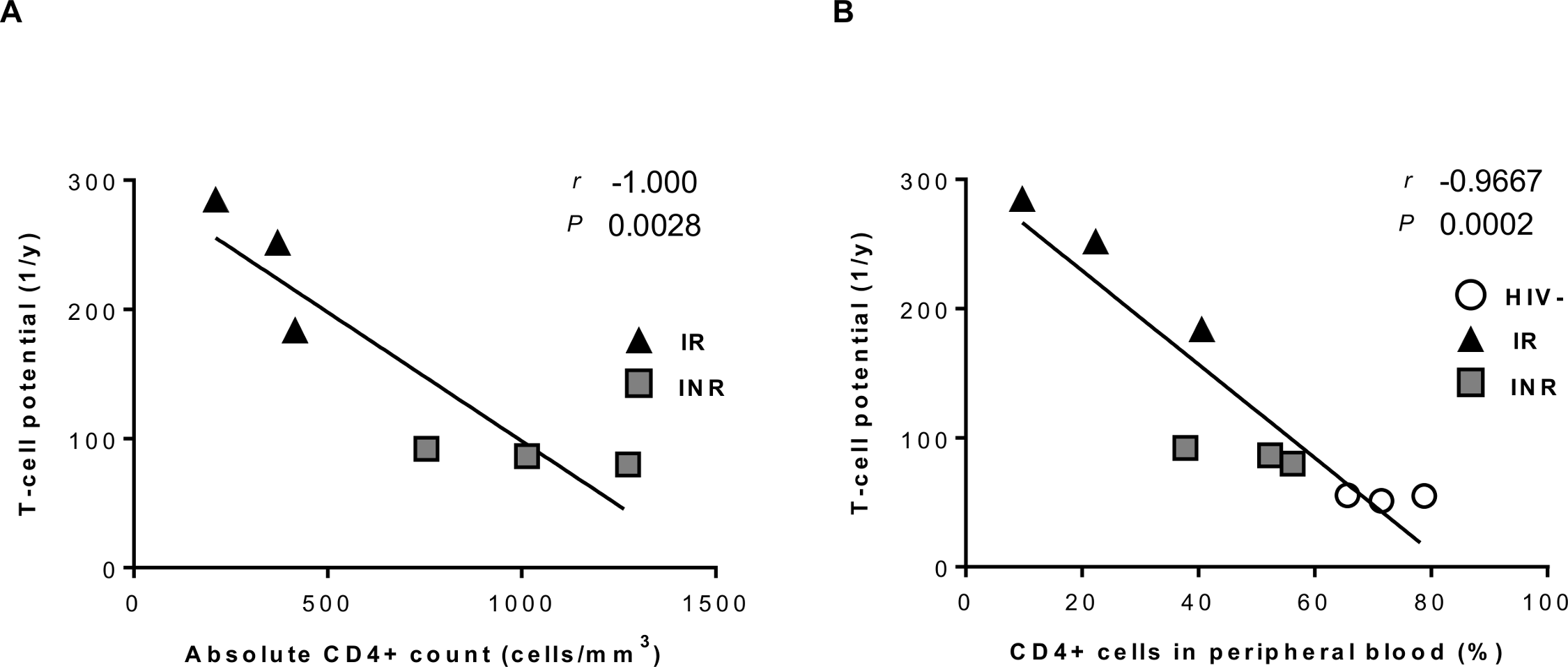

Supplement: S1 Fig — Correlation of T-cell potential with absolute CD4+ count (A) and the percentage of circulating CD4+ cells (B). Spearman’s rank correlation analysis was used to determine the slope. P values <0.05 were considered significant. (TIF) [file ppat.1005571.s001.tif]

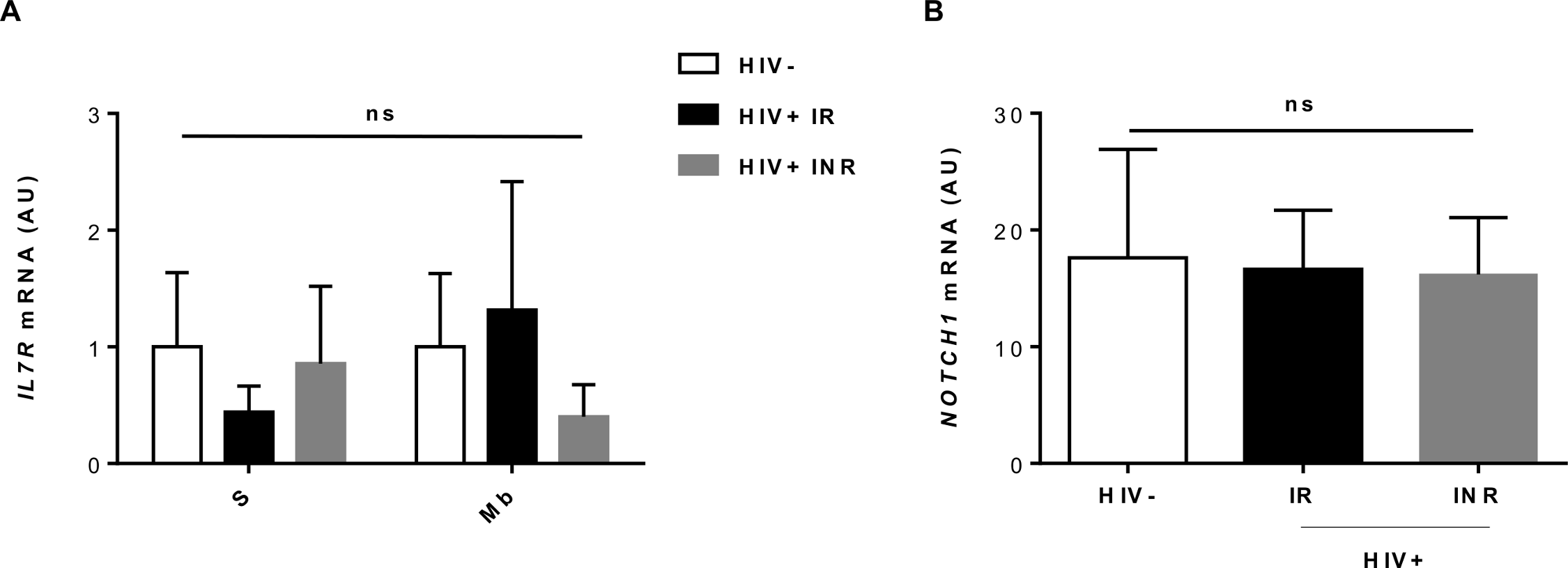

Supplement: S2 Fig — (A) RT-qPCR analysis of soluble (S) and membrane-bound (Mb) IL7RA (HIV-, n = 3; HIV-positive IRs, n = 10; HIV-positive INRs, n = 7). Means and standard errors are shown. The Kruskal-Wallis test was used to assess differences between groups and Wilcoxon test was used to compare sIL7RA to mbIL7RA for each group. NS for P>0.05. (B) RT-qPCR analysis of NOTCH1 mRNA (HIV-, n = 4; HIV-positive IRs, n = 4; HIV-positive INRs, n = 5). Means and standard errors are shown. The Kruskal-Wallis test was used to assess differences between groups. NS for P>0.05. (TIF) [file ppat.1005571.s002.tif]

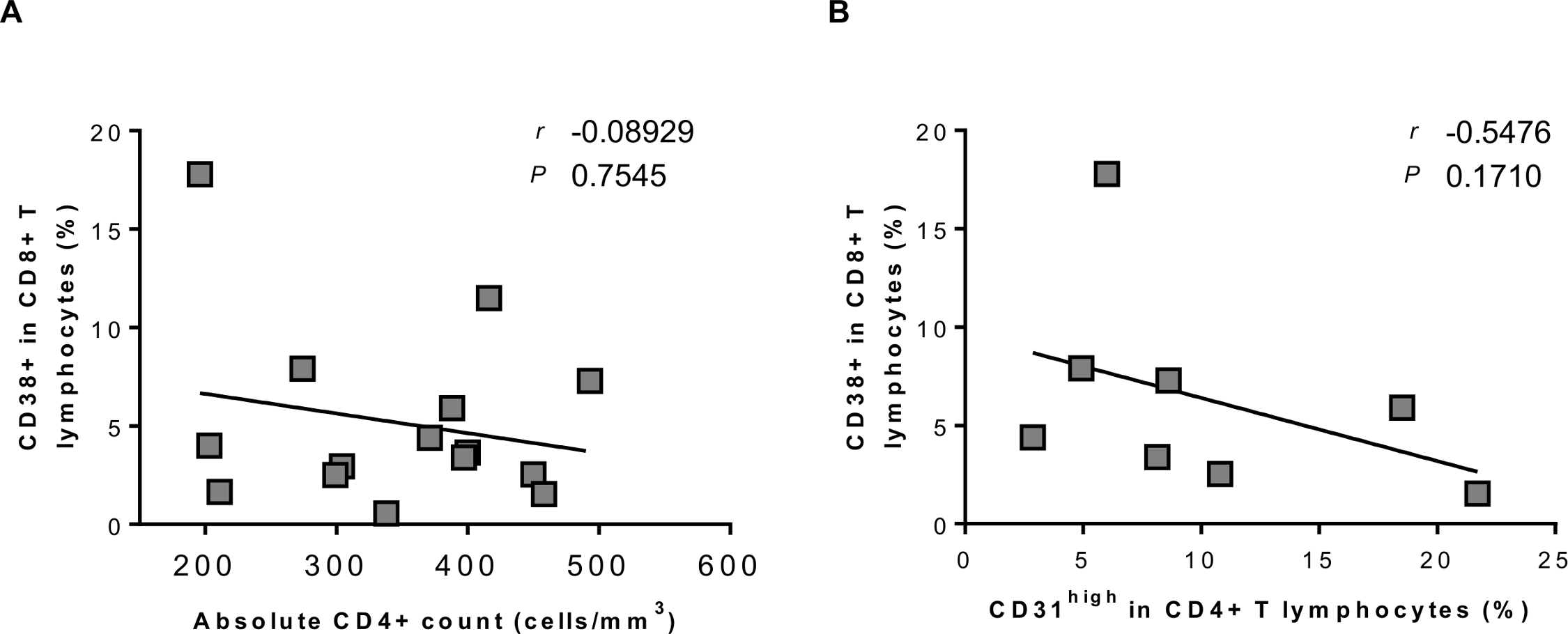

Supplement: S3 Fig — (A, B) The percentage of CD38high cells among CD8+ T lymphocytes indicates the degree of immune activation in correlation along peripheral absolute CD4+ T-cell count (A) and CD4+ RTEs percentage in peripheral blood (B). Spearman’s rank correlation analysis was used to determine the slope. P values <0.05 were considered significant. (TIF) [file ppat.1005571.s003.tif]

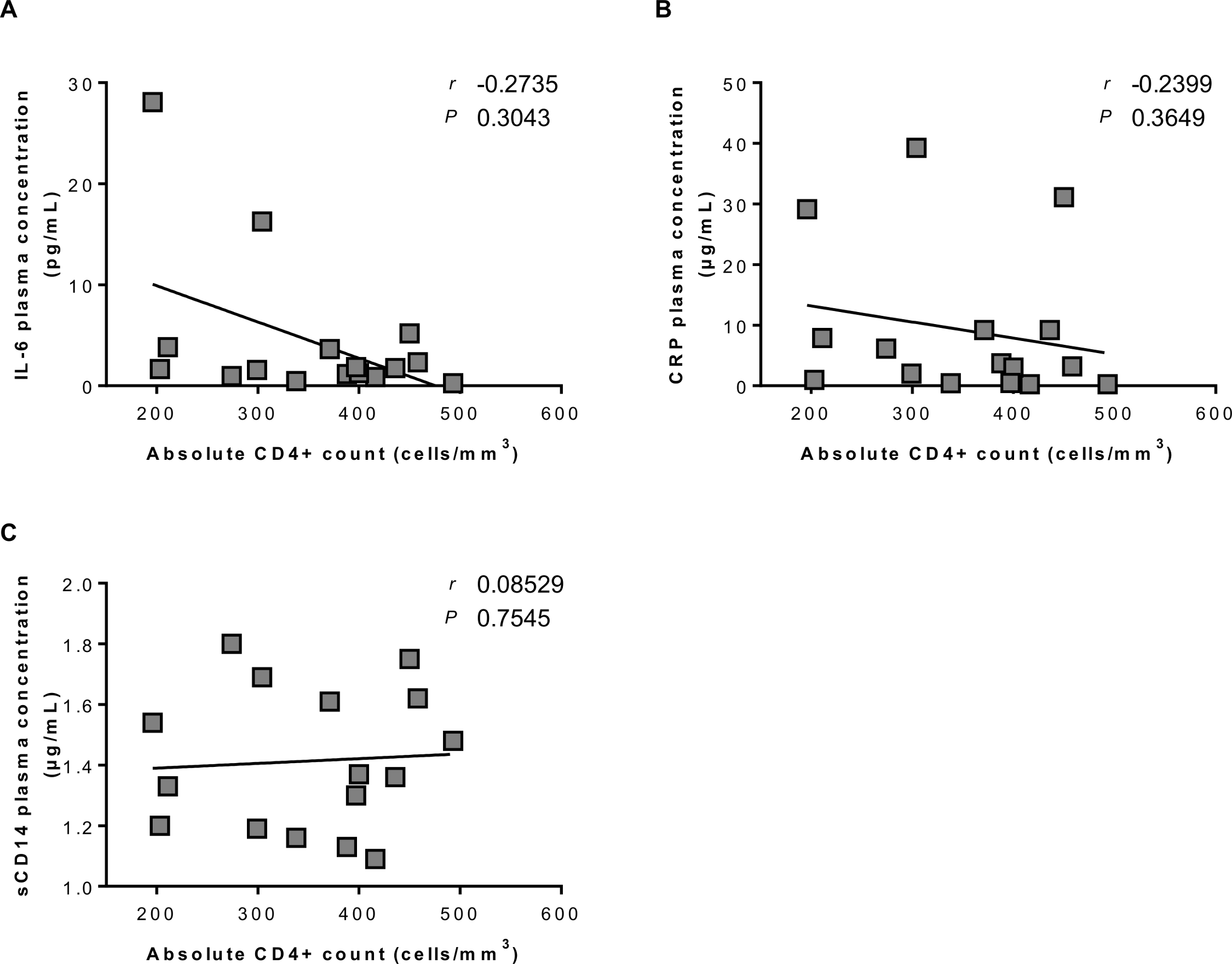

Supplement: S4 Fig — (A) Plasma concentrations of IL-6 (A), CRP (B), and sCD14 (C) indicate the degree of inflammation. Spearman’s rank correlation analysis was used to determine the slope. P values <0.05 were considered significant. (TIF) [file ppat.1005571.s004.tif]

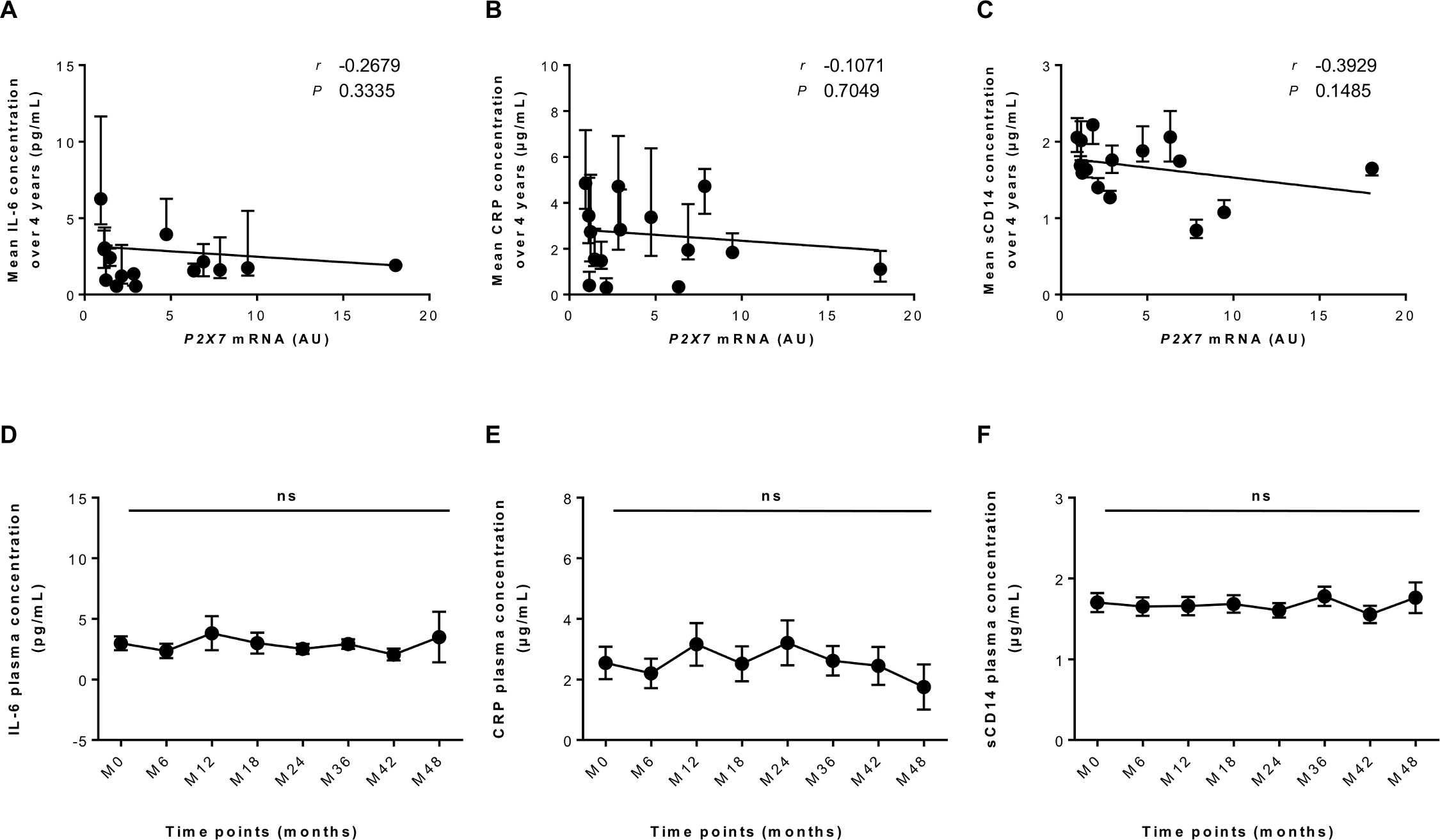

Supplement: S5 Fig — (A, B, C) Correlations of the mean concentrations of 8 available determinations per patient of IL-6 (A), CRP (B) and sCD14 (C) with P2X7 mRNA levels (HIV-positive, n = 15). Spearman’s rank correlation analysis was used to determine the slope. P values <0.05 were considered significant. (D, E, F) Analysis of IL-6 (D), CRP (E) and sCD14 (F) concentrations over the last four years (2 time points per year) in 15 HIV-infected patients. Means and standard errors are shown. Friedman’s test was used for statistical analysis. P values <0.05 were considered significant. (TIF) [file ppat.1005571.s005.tif]
